# Multiattribute Analysis of *Trichomonas vaginalis* Diagnostics and Its Correlation with Clinical Complaints and Contraceptive Methods in a Symptomatic Egyptian Cohort

**DOI:** 10.1155/2021/5525095

**Published:** 2021-04-29

**Authors:** Marwa M. I. Ghallab, Doaa Alaa, Salwa M. Morsy

**Affiliations:** ^1^Medical Parasitology, Faculty of Medicine, Kafrelsheikh University, Egypt; ^2^Department of Obstetrics and Gynecology, Faculty of Medicine, Cairo University, Egypt; ^3^Department of Medical Parasitology, Faculty of Medicine, Cairo University, Egypt; ^4^Faculty of Medicine, Modern University for Technology and Information, Cairo, Egypt

## Abstract

**Background:**

*Trichomonas vaginalis* (*T. vaginalis*) infection has been long considered among the sexually transmitted diseases that possesses a clear effect on women's health especially in the childbearing period.

**Methods:**

A 234 females of age range 18-45 years old attending the Gynecology and Obstetrics Outpatient Clinic of Kasr El Aini Hospitals were enrolled in a cross-sectional study. The taken vaginal swabs were subjected to wet mount microscopy, Giemsa stain, modified Diamond's culture, and nested polymerase chain reaction (nPCR) amplification. Multiattribute and analytical hierarchy processes were conducted to detect laboratory utility. Univariate and multivariate analyses were done to detect the multiple risk factors that may be associated with *Trichomonas* infection.

**Results:**

Based on nPCR, the prevalence of trichomoniasis was 26.9%. Wet mount, Giemsa stain, and culture showed 100% specificity but of low sensitivity (28.57%, 28.57%, and 57.14%, respectively). On the multivariate analysis, nPCR showed the highest rank for diagnostic performance and culture had the lowest rank. For univariate analysis, there was a significant correlation between *T. vaginalis* infection and vaginal discharge, burning sensation, dyspareunia, and the use of intrauterine device (IUD) (*P* value < 0.05).

**Conclusion:**

The routine screening of trichomoniasis using nPCR was reliable, sensitive, and specific. Also, it could financially be considered a more suitable option in batch screening. Significant higher rates of infection were reported among IUD users compared to condom or hormonal-based methods.

## 1. Background

Trichomoniasis, the commonest sexually transmitted infectious disease worldwide that mostly occurs in women in the childbearing period, is caused by *T. vaginalis*, an anaerobic flagellated urogenital parasite [1]. Though it is a curable infection, it can lead to adverse health consequences [2]. According to WHO, there are about 143 million new cases annually [3].

In male, infection is mostly asymptomatic or it may be just tiny urethral discharge [4]. Females' infection may be asymptomatic, or it has been associated with a wide range of clinical sequels including symptoms of vaginitis, urethritis, cervicitis, pelvic inflammatory disease, ectopic pregnancy, or even infertility [5, 6].

Recently, *T. vaginalis* infection has been linked to risk increase in acquisition of HIV and cancer. Heavy untreated infection during pregnancy has been associated with premature rupture of membranes, preterm delivery, and low birth weight [7].

Many risk factors may affect the epidemiology of trichomoniasis including age, residence, sociocultural level, education, marital state and the type of contraception method used (IUD/condom usage or other used contraceptives), presence and type of vaginal discharge, the used drug, and history of other sexually transmitted infections [8].

Diagnosis of *T. vaginalis* infection is difficult, as its symptoms mimic those of other sexually transmitted infections and it needs a confirmatory test. The diagnosis is made by conventional direct microscopic wet mount, staining, or culture preparation. Other diagnostic methods include immunological detection, or nucleic acid-based assays were also used [9].

This study was aimed primarily at determining the prevalence of *T. vaginalis* infection and the possible associated risk factors especially its relation to the contraception methods among women attending the Gynecology and Obstetrics Outpatient Clinic at Kasr Al Aini School of Medicine, Cairo, Egypt, using conventional direct wet mount microscopic examination, Giemsa stain, Diamond's media culture, and nested PCR assay. Additionally, multiattribute analysis and analytical hierarchy process of the diagnostic methods based on their diagnostic yield and lab utility were a secondary outcome.

## 2. Material and Methods

### 2.1. Study Design

After ethical approval by the ethical committee of Faculty of Medicine, Kafrelsheikh University, an informed consent was obtained from each patient.

A cross-sectional study was conducted over 240 females attending the Gynecology and Obstetrics Outpatient Clinic of Kasr El Aini Hospitals from May 2018 to April 2019. But 6 samples were spilled during preparation so statistics was done over 234 samples only.

Females incorporated in the study were married from 18 to 45 years old on contraception, complaining of any gynecological complaints like vaginal discharge, burning sensation, pruritus vulvae, dyspareunia, or dysuria.

A designed questionnaire was done to record personal, contraception, and clinical history including the absence of any medical diseases.

Women who are pregnant, are menopausal, had recent vaginal douches, or had coitus in the past 2 days before the sampling and women with past history of administration of either antibiotics, antiprotozoal treatment, or steroid in the past two weeks were excluded.

### 2.2. Sample Collection and Processing

Each participant was examined by a sterile nonlubricated speculum, and three vaginal swabs were taken from the posterior fornix of the vagina by a commercially prepared sterile swab; all samples were examined as follows:

1. Wet mount microscopy and Giemsa staining

One swab was kept in 3 ml sterile phosphate-buffered saline (PBS) for both wet mount microscopy where slides were examined immediately for motile trophozoite and Giemsa staining.

(2) Culture (modified Diamond's medium)

The second swab was inoculated immediately into the culture tube containing culture medium according to [6].

(3) Nested PCR assay

The third swab was immediately eluted in phosphate-buffered saline and preserved in -20°C until further molecular workup.

Extraction of the genomic DNA was done using DNA extraction: DNA was extracted using the QIAamp® DNA Mini Kit according to the manufacturer's instructions. The purity and concentration of the extracted DNA were measured using a fluorometer. Screening of all the samples was done using nPCR targeting the Actin gene according to [10], the used primers are tabulated in Table 1, and the cycling conditions were as follows: an initial denaturing step of 5 minutes at 95°C followed by 35 cycles of denaturation at 95°C for 30 s, annealing at 55°C for the primary reaction and 52°C for the nested one for 45 s, and extension at 72°C The amplified products were visualized with 1.5% agarose gel electrophoresis and visualized under ultraviolet transilluminator.

### 2.3. Statistical Analysis

Data were coded, tabulated, and analyzed using the statistical package SPSS version 25, and data were considered statistically significant if *P* value was <0.05. Sensitivity, specificity, positive predictive value, negative predictive value, accuracy, and kappa agreement were calculated to test the diagnostic yield of the 3 used tests in relation to nPCR. The multiattribute utility theory and analytical hierarchy process were both used to evaluate the lab utility and the diagnostic performance of the used diagnostic procedure according to [11].

## 3. Results

### 3.1. Results of the Wet Mount and Giemsa in relation to nPCR

**Figure 1:**
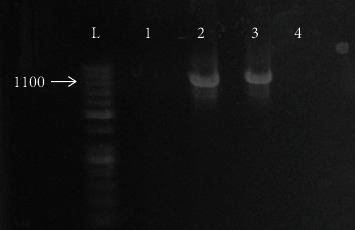
Gel electrophoresis of amplified DNA products of the Actin gene. L: 100 bp ladder. Lanes 1 and 2: negative and positive controls, respectively. Lanes 3 and 4: positive and negative samples, respectively.

A total of 234 women were incorporated in our study. Examination of their vaginal swabs using wet mount and Giemsa stain revealed 18 (7.7%) positive samples, culture succeeded to detect positivity in 36 (15.4%), while nPCR detected 63 positive samples (26.9%) (Figure 1 and Table 2).

The performance of the 3 diagnostic techniques was compared using results of the nPCR assay as the gold standard (Table 3).

Wet mount microscopy and Giemsa staining showed low sensitivity (28.57%) and substantial agreement (0.8) compared to the high sensitivity of the culture method (57.14%) and perfect agreement (0.88), but all of them showed high specificity (100%).

### 3.2. Results of Multiattribute and Analytical Hierarchy Process

Analyzing the ranked results using the analytical hierarchy process to determine the best laboratory testing option revealed that nPCR had the highest rank (11.9), followed by wet mount microscopy method (9.8), then the Giemsa stain method (9.1), and lastly the culture method (5.4) (Table 4).

### 3.3. Univariate and Multivariate Analysis of Multiple Risk Factors

The majority of the cases were complaining of vaginal discharge (61) (26%), and univariate analysis of study variables revealed that there was a statistically significant association between *T. vaginalis* infection and the presence of vaginal discharge, burning sensation, and dyspareunia (*P* value = 0.001, 0.001, and 0.004, respectively) (Table 5).

Regarding contraceptive methods, there was a statistically significant correlation between trichomoniasis and the use of contraception generally (*P* ≤ 0.001) and IUD specially (*P* ≤ 0.001).

These variables were subjected for multivariate analysis using logistic regression that showed statistical correlation between the probability of trichomoniasis infection and the presence of vaginal discharge (*P* ≤ 0.001, OR = 2.421, CI = 0.793-1.278), burning sensation (*P* ≤ 0.001, OR = 1.406, CI = 0.228-0.431), and dyspareunia (*P* = 0.026, OR = 0.376, CI = 0.173–0.374), besides the use of IUD (*P* ≤ 0.001, OR = 0.136, CI = 0.064-0.291).

## 4. Discussion

Till today, little emphasis has been laid on the importance of decreasing the rates of *T. vaginalis* infection even though it has been associated with HIV acquisition [12].

The routine attempts targeting the detection and treatment of trichomoniasis have gained great interest owing to its recent association with the adverse gynecological and obstetric outcomes, infertility and HIV infection [13]. However, *Trichomonas* infection attracts little attention in developing countries in spite of its adverse sequel on the reproductive health; this may be because most infection is asymptomatic in addition to the burden of the poor diagnostic techniques [14].

In the present study, the prevalence of *T. vaginalis* infection among study groups using nPCR was indeed high, 26.9%.

Though this could be due to our selected study population, it is also an indication of the long existence of the infection in our community; the lack of awareness and routine laboratory detection may have compounded the problem in Egypt; the prevalence varies between 5% and 91.3% [2].

The disparity of the reported prevalence may be attributed to the variations in the target population under study, sociocultural factors, age, menstrual cycle phase, the coinfection with other sexually transmitted infections, specimen type, collection and processing, available diagnostic techniques, and test interpretation [15].

Although wet mounts/staining and culture showed high specificity (100%), the first two methods showed poor sensitivity and the culture method showed moderate sensitivity as many cases may escape diagnosis. This came in accordance with other studies [15, 16]. The decreased sensitivity may be due to the need of multiple sampling which is surely time-consuming and needs more than one expertise.

Wet mount/staining showed moderate agreement while the culture method showed substantial agreement with nPCR.

This was in disagreement with [13] who reported a poor agreement between PCR assay as the gold standard and the wet mount microscopy.

In the present study, multiattribute analysis ranked nPCR with the highest rank, followed by wet mount microscopy method, then the Giemsa stain method, and lastly the culture method.

Though wet mount is the most widely used diagnostic method because it is simple, rapid, practical, and relatively cheap, there is a clear evidence that it is an unreliable and unsatisfactory screening test as it is time-consuming and needs an expert; due to the difficulty in differentiating it from the nucleus of vaginal epithelial cell or neutrophil, samples should be examined within 20 minutes and it also depends on the severity of infection [17]. Moreover, it is clearly documented that the modified Diamond culture could serve as a laboratory testing option but it is expensive and time-consuming, and it needs incubators. Additionally, it is not available in all laboratories [4].

Based on the obtained results, nPCR could serve as an attractive diagnostic option as it can be used in batch sample diagnosis and it is an efficient strain typing technique that could aid in understanding the nature and extent of genetic diversity of *T. vaginalis* aiding in the epidemiological control programs.

These study results showed that there was a statistical correlation between trichomoniasis and vaginal discharge, burning sensation, and dyspareunia. This was in accordance with other studies in Nigeria [17], Aethiopia [18], Brazil [19], and Egypt [15].

It was found that there was a statistical correlation between the use of contraception method and trichomoniasis, and multivariate analysis revealed a statistical association with the use of IUD compared to both condom and hormonal methods. This was in accordance with ([18, 20] [21], but others reported that hormonal contraception is strongly associated with trichomoniasis [22].

Contrary to ours, [19] found no association between trichomoniasis prevalence and use of contraceptive nor the presence of vaginal discharge.

The limitation to this study is that we need to include asymptomatic women in further studies as one-third of infection could be asymptomatic. Moreover, genotypic differentiation of different *Trichomonas* isolates and its correlation to symptomatic and asymptomatic cases should be investigated in the future work.

## 5. Conclusion

*T. vaginalis* had a high recovery rate in our community though it is still neglected. So, great efforts need to be integrated in the screening programs of reproductive health and family planning.

More studies should be done on a larger sample size, different geographical areas, and sociocultural levels. Great attention should be paid to assess the disease in the asymptomatic and pregnant female and its sequel on the maternal and fetal outcomes.

Though wet mount, staining, and culture methods had many advantages, they had many drawbacks. So, the PCR method offers a reliable, sensitive, specific screening tool.

## Figures and Tables

**Table 1:** The used primers' sequences.

	Primers	Sequence	Annealing temp.
1^ry^ PCR	Tv8S	5′-TCTGGAATGGCTGAAGAAGACG-3′	55°C
	Tv9R	5′-CAGGGTACATCGTATTGGTC-3′	

2^ry^ PCR	Tv10S	5′-CAGACACTCGTTATCG-3′	50°C
	Tv11R	5′-CGGTGAACGATGGATG-3′	

**Table 2:** Results of the used diagnostic methods.

		PCR (total *n* = 234)	
		Positive (*n* = 63)	Negative (*n* = 171)
Wet mount	+ve	18 (7.7%)	0 (0%)
	-ve	45 (19.2%)	171 (69.3%)

Giemsa stain	Yes	18 (7.7%)	0 (0%)
	No	45 (19.2%)	171 (69.3%)

Culture	Yes	36 (15.4%)	0 (0%)
	No	27 (11.5%)	171 (69.3%)

**Table 3:** The diagnostic performance of the used diagnostic methods.

	Wet mount	Giemsa stain	Culture
Sensitivity	28.57%	28.57%	57.14%
Specificity	100.00%	100.00%	100.00%
Positive predictive value	100.00%	100.00%	100.00%
Negative predictive value	79.17%	79.17%	86.36%
Kappa	0.8	0.8	0.88

Key for kappa: <0: poor agreement, 0.01-0.20: slight agreement, 0.21-0.40: fair agreement, 0.41-0.60: moderate agreement, 0.61-0.8: substantial agreement, and 0.81-1.00: almost perfect agreement.

**Table 4:** Analytical hierarchy process of the overall ranking of different diagnostic techniques under study.

Method	Evaluation item							Total
	Performance		Costs	Ease of		Batch ability	Species identification	
	Sensitivity	Specificity		Use	Interpretation			
Priority value Rank of method	0.35	0.35	0.95	0.9	0.15	0.5	0.7	
Wet mount	1	4	4	4	1	1	0	9.8
Giemsa stain	1	4	3	3	2	3	0	9.1
Culture	2	4	1	1	3	2	0	5.4
nPCR	4	4	2	2	4	4	4	11.9

**Table 5:** Clinical complaints and contraception methods used in relation to nPCR results.

		Total (*n* = 234)		*P* value
		Positive (*n* = 63)	Negative (*n* = 171)	
Discharge	Yes	61 (26%)	83 (35.5%)	0.001
	No	2 (0.8%)	88 (37.7%)	

Itching	Yes	20 (8.5%)	72	0.098
	No	43 (18.4%)	99	

Burning sensation	Yes	56	73	0.001
	No	7	98	

Dyspareunia	Yes	27	108	0.004
	No	36	63	

Contraception	Yes	56	97	0.001
	No	7	74	

Intrauterine device (IUD)	Yes	25	16	≤0.001
	No	10	95	

Pills	Yes	13	31	0.663
	No	11	20	

Condom	Yes	2	9	0.614
	No	2	0	

## Data Availability

All data is available from the corressponding author upon reasonable reasons.
